# Hepatitis C Virus Induces the Ubiquitin-Editing Enzyme A20 via Depletion of the Transcription Factor Upstream Stimulatory Factor 1 To Support Its Replication

**DOI:** 10.1128/mBio.01660-19

**Published:** 2019-07-23

**Authors:** Jiyoung Lee, Stephanie T. Chan, Ja Yeon Kim, Jing-hsiung James Ou

**Affiliations:** a Department of Molecular Microbiology and Immunology, Keck School of Medicine, University of Southern California, Los Angeles, California, USA; Virginia Polytechnic Institute and State University; University of California San Diego; University of Alabama at Birmingham School of Medicine

**Keywords:** A20 ubiquitin enzyme, HCV IRES, USF-1 transcription factor, hepatitis C virus

## Abstract

Tumor necrosis factor alpha-induced protein 3 (TNFAIP3), also known as A20, is a ubiquitin-editing enzyme capable of ubiquitination or deubiquitination of its target proteins. In this study, we show that hepatitis C virus (HCV) infection could induce the expression of A20 via the activation of the A20 promoter. The induction of A20 by HCV coincided with the loss of upstream stimulatory factor 1 (USF-1), a transcription factor known to suppress the A20 promoter. The role of USF-1 in the regulation of the A20 promoter in HCV-infected cells was confirmed by the chromatin immunoprecipitation (ChIP) assay, and its depletion was apparently mediated by proteasomes, as USF-1 could be stabilized by the proteasome inhibitor MG132 to suppress the A20 expression. As the overexpression of A20 enhanced the replication of HCV and the silencing of A20 had the opposite effect, A20 is a positive regulator of HCV replication. Our further studies indicated that A20 enhanced the activity of the HCV internal ribosome entry site (IRES). In conclusion, our results demonstrated that HCV could induce the expression of A20 via the depletion of USF-1 to enhance its replication. Our study provided important information for further understanding the interaction between HCV and its host cells.

## Abstract (precis)

Hepatitis C virus establishes chronic infection in approximately 85% of the patients whom it infects. However, the mechanism of how HCV evades host immunity to establish persistence is unclear. In this report, we demonstrate that HCV could induce the expression of the ubiquitin-editing enzyme A20, an important negative regulator of the tumor necrosis factor alpha (TNF-α) and NF-κB signaling pathways. This induction of A20 enhanced HCV replication as it could stimulate the HCV IRES activity to enhance the translation of HCV proteins. The induction of A20 was mediated by the depletion of USF-1, a suppressor of the A20 promoter. Our study thus provides important information for further understanding the interaction between HCV and its host cells.

## INTRODUCTION

Chronic infection by hepatitis C virus (HCV) is associated with severe liver diseases, including steatosis, cirrhosis, and hepatocellular carcinoma. The estimated number of patients with chronic HCV infection is approximately 70 million worldwide, and up to 4 million people become infected by HCV every year (1). HCV is a hepatotropic virus. Its infection is difficult to detect during the acute phase as it is mostly asymptomatic. A majority (∼85%) of HCV patients fail to clear HCV infection and become chronic HCV carriers. How HCV evades host immunity to establish chronic infection is an interesting question that remains largely unclear.

Host immune responses to microbial infections such as viral infections are tightly regulated, as a failure to keep the immune homeostasis can be detrimental to the host. Many of these regulations are achieved through the timely control of the assembly and disassembly of receptor signaling complexes via posttranslational modifications of their component proteins (2). Ubiquitination is one such important modification. For example, the tumor necrosis factor alpha (TNF-α) receptor 1 (TNFR1)/NF-κB signaling is controlled by the ubiquitination of receptor-interacting serine/threonine protein kinase 1 (RIPK1) and IκB, two important regulatory factors of the TNFR1/NF-κB signaling pathway. The TNF-α-induced protein 3 (TNFAIP3), also known as A20, is a ubiquitin-editing enzyme with dual functions. The ovarian tumor (OTU) domain located at its N terminus has a deubiquitinating activity, and the zinc finger-containing domain located at its C terminus has an E3 ubiquitin ligase activity. A20 requires the E3 ligase Itch and the RING finger protein 11 (RNF11) to form the A20 ubiquitin-editing complex (3). Although the induction of A20 follows the activation of NF-κB, once induced A20 negatively regulates the activity of NF-κB by removing the K63-linked ubiquitin chains from RIPK1 and also adding the K48-linked ubiquitin chains to RIPK1, leading to its degradation and the control of the NF-κB activity (4).

HCV belongs to the flavivirus family and has an ∼9.6-kb positive-stranded RNA genome. Its genome encodes a polyprotein that is slightly longer than 3,000 amino acids. During its translation, this HCV polyprotein is cleaved by cellular and viral proteases to generate 10 mature protein products. The translation of the HCV polyprotein is cap independent and mediated by an internal ribosome entry site (IRES) that encompasses most of its 5′ untranslated region and the first 9 amino acids of its coding sequence (5, 6).

We had previously studied the host innate immune response to HCV infection and demonstrated that HCV infection could induce TNF-α via the activation of Toll-like receptor (TLR) 7/8 and NF-κB (7). This led to the autocrine activation of TNFR1 and prevented the depletion of type I interferon receptor 2 (IFNAR2) by HCV. To further understand the interaction between HCV and the TNF-α signaling, we examined the possible effect of HCV on A20. Our results indicated that HCV could induce the expression of A20 to enhance its replication. This induction of A20 by HCV was mediated by the depletion of upstream stimulatory factor 1 (USF-1), a transcriptional factor that suppresses the A20 promoter and the expression of A20.

## RESULTS

### HCV induces A20 expression.

**FIG 1:**
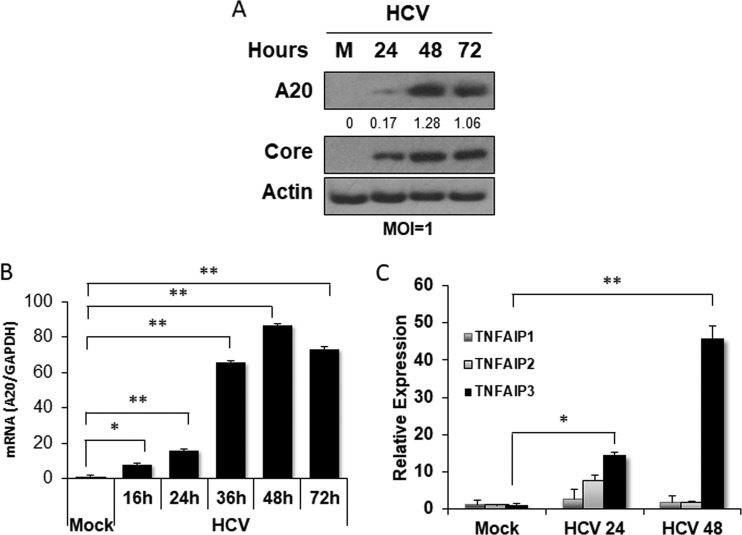
Induction of A20 by HCV infection. Huh7 cells were infected with HCV (MOI = 1) and lysed at the time points indicated for analysis. (A) Immunoblot analysis of cell lysates. A20 and the HCV core protein were analyzed. Actin served as the loading control. (B) Real-time RT-PCR (qRT-PCR) analysis of the A20 mRNA. The experiment was conducted in triplicate, and the results were normalized against the GAPDH mRNA and then against the mock-infected cells. (C) qRT-PCR analysis of TNFAIP mRNAs. Huh7 cells were infected with HCV at an MOI of 1 and lysed at 24 and 48 h postinfection for the isolation of RNA for qRT-PCR. TNFAIP3 is a different name for A20. The experiments were conducted in triplicate, and the results were normalized against GAPDH mRNA. *, *P* < 0.05; **, *P* < 0.001.

A20 is a key regulatory factor that suppresses the TNF-α-induced activation of NF-κB (8, 9). To determine the possible effect of HCV on A20, we infected Huh7 hepatoma cells with a cell culture-adapted variant of the HCV JFH1 strain using a multiplicity of infection (MOI) of 1. Cells were then lysed at different time points after the infection for immunoblot analysis. As shown in Fig. 1A, a slight increase in A20 protein expression was observed at 24 h postinfection. This increase became highly prominent at 48 h and 72 h. The analysis of the A20 mRNA levels by real-time RT-PCR revealed a similar result. The increase of the A20 mRNA level was detectable at 16 h postinfection and peaked at 48 h followed by a slight decrease at 72 h (Fig. 1B). The effect of HCV on A20 (i.e., TNFAIP3) was specific, as HCV was able to only slightly and transiently increase the expression of TNFAIP1 and TNFAIP2, two other TNF-α-induced proteins, at 24 h postinfection but not at 48 h (Fig. 1C). Thus, the results shown in Fig. 1 indicated that HCV could specifically induce the expression of A20, likely at the transcriptional step.

### HCV-induced A20 expression is regulated by USF-1.

**FIG 2:**
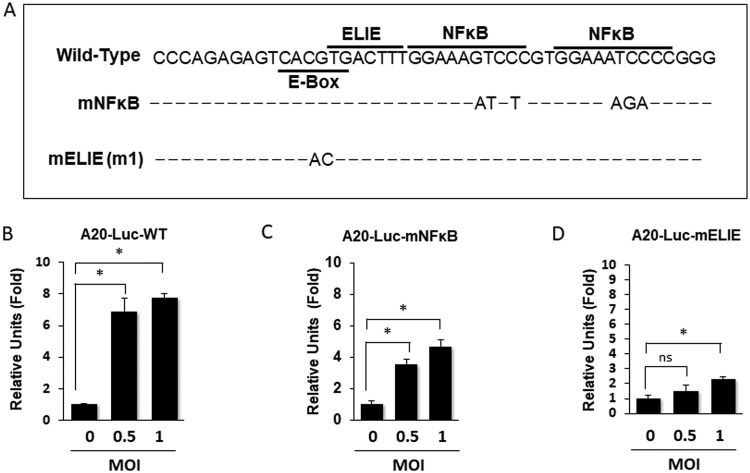
Analysis of the effect of HCV on the A20 promoter. (A) Schematic representation of the A20 promoter. The locations of the E-box, the ELIE motif, and the NF-κB binding sites are highlighted. The mNFκB A20 promoter construct contains mutations in the two NF-κB binding sites, and the mELIE (m1) A20 promoter construct contains mutations in the overlapping E-box and ELIE sequence. (B to D) Huh7 cells were transfected with the firefly luciferase reporter plasmid that was under the expression control of the wild-type A20 promoter (B), the mNFκB A20 promoter (C), or the mELIE (m1) promoter (D). The plasmid pCMV-renilla was included in the cotransfection to serve as the internal control to monitor the transfection efficiency. Twenty-four hours after DNA transfection, cells were infected with HCV at MOIs of 0.5 and 1 for 48 h and then lysed for analysis of the luciferase activities using the dual-luciferase assay kit (Promega). The experiments were conducted in triplicate, and the firefly luciferase results were normalized against renilla luciferase results for the calculation of relative luciferase units. The results represent the mean ± SEM from two independent experiments. *, *P* < 0.05; ns, not significant.

The A20 promoter contains an E-box that overlaps with an elongation inhibitory element (ELIE) and two juxtaposed NF-κB binding sites (Fig. 2A) (10, 11). The overlapping E-box and ELIE negatively regulate the expression of A20. The E-box is bound by upstream stimulatory factor 1 (USF-1), which interacts with the dichloro-1-β-d-ribofuranosylbenzimidazole (DRB) sensitivity-inducing factor (DSIF) to halt the RNA elongation by RNA polymerase II, keeping the A20 expression in check (10, 11). To determine whether HCV could indeed transcriptionally activate the expression of A20, we tested the possible effect of HCV on the A20 promoter. We used the plasmid containing the A20 promoter sequence (−240 to −10) fused to the coding sequence of the firefly luciferase reporter (A20-Luc-WT). This plasmid was transfected into Huh7 cells, which were then infected with HCV. As shown in Fig. 2B, HCV was able to activate the A20 promoter at 48 h postinfection, supporting the notion that HCV could transcriptionally activate the expression of A20. To further understand how HCV activated the A20 promoter, we examined the possible effect of HCV on the A20 promoter that carried mutations in either the E-box/ELIE (A20-Luc-mELIE) or the NF-κB binding sites (A20-Luc-mNFκB) (Fig. 2A). These mutations in E-box/ELIE and NF-κB binding sites had previously been shown to abolish the binding of USF-1 and NF-κB, respectively, to the promoter of A20 (10, 11). As shown in Fig. 2C, mutations introduced into the NF-κB sites reduced but did not abolish the effect of HCV on the A20 promoter. In contrast, mutations introduced into the overlapping E-box/ELIE sequence greatly diminished the ability of HCV to activate the A20 promoter. These results indicated that the E-box/ELIE site played a more critical role than the NF-κB binding sites in mediating the activation effect of HCV on the A20 promoter.

**FIG 3:**
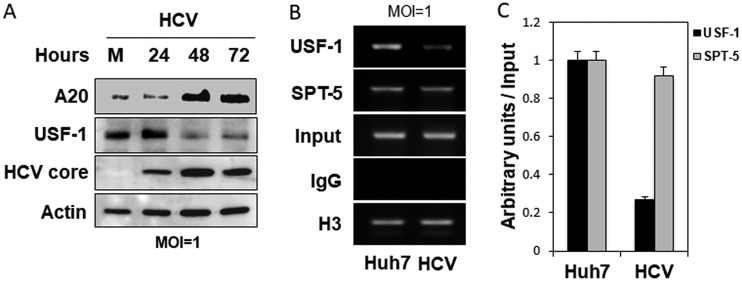
Depletion of USF-1 by HCV. (A) Immunoblot analysis of A20, USF-1, and HCV core. Huh7 cells were infected with HCV (MOI = 1) and lysed at the time points indicated for immunoblot analysis. Actin served as the loading control. (B) ChIP analysis of USF-1 binding to the A20 promoter. Huh7 cells were either mock infected or infected with HCV (MOI = 1) for 24 h. Cells were then fixed with formaldehyde for ChIP analysis as described in Materials and Methods. Histone H3 protein and SPT-5 served as the internal positive controls, and the control antibody IgG was used as the negative control. (C) Quantification of the results shown in panel B, which were measured by ImageJ and normalized against the input control. The results represented the average from 2 independent experiments.

The E-box/ELIE site is recognized by the transcription factor USF-1. As this site mediates the activation effect of HCV on the A20 promoter, we examined the possible effect of HCV on the expression of USF-1 by conducting the immunoblot analysis. As shown in Fig. 3A, HCV reduced the expression level of USF-1 in a time-dependent manner up to 72 h after infection, the study endpoint. The decrease in the USF-1 level coincided with the increase in the A20 level. The binding of USF-1 to the E-box/ELIE was further analyzed by the chromatin immunoprecipitation (ChIP) assay. As shown in Fig. 3B, HCV infection reduced the binding of USF-1 to the A20 promoter. In contrast, HCV had little effect on the binding of SPT-5, a transcription elongation factor and a component of DSIF, to the A20 promoter. The ChIP results for USF-1 and SPT-5 were quantified and normalized against that of the internal control histone H3 (Fig. 3C). Taken together, the results shown in Fig. 2 and 3 indicated that HCV likely reduced the expression level of USF-1 to activate the A20 promoter for the induction of A20 expression.

### HCV induced proteasomal degradation of USF-1 and the induction of A20.

**FIG 4:**
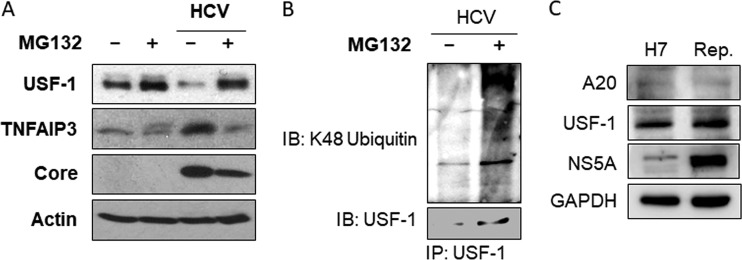
Effect of MG132 and HCV subgenomic RNA replicon on USF-1. (A) Huh7 cells were either mock infected or infected with HCV at an MOI of 1 for 36 h followed by 12 h of treatment with either the vehicle dimethyl sulfoxide (DMSO) or MG132 (10 μM). Cell lysates were then subjected to immunoblot analysis with the antibodies indicated. (B) Huh7 cells transfected with the HA-ubiquitin plasmid were infected with HCV and then treated with DMSO or MG132 for 6 h. Cells were then lysed for immunoprecipitation using the anti-USF-1 antibody followed by immunoblot analysis. (C) Control Huh7 cells (H7) or HCV replicon cells (Rep.) were lysed for immunoblot analysis. No significant effect of replicon on A20 or USF-1 was observed.

To understand how HCV might reduce the protein level of USF-1, we treated control and HCV-infected cells with the proteasome inhibitor MG132. The treatment of the control mock-infected cells with MG132 led to a slight increase of the USF-1 level and no significant decrease of the A20 level (Fig. 4A). This result indicated that proteasomes might play only a limited role in maintaining the steady-state level of USF-1 in mock-infected cells. In agreement with the results shown in Fig. 3A, HCV infection reduced the level of USF-1 and increased that of A20. The reduction of USF-1 by HCV was restored by MG132, indicating that HCV likely triggered the degradation of USF-1 via the proteasomal pathway. Also, in agreement with the results shown in Fig. 3A, the restoration of the USF-1 level by MG132 in HCV-infected cells reduced A20 back to the basal level, confirming a suppressive role of USF-1 in A20 expression. To further confirm that USF-1 was indeed degraded by the ubiquitin-proteasome pathway, we also analyzed whether the inhibition of proteasomes with MG132 would lead to the increase of ubiquitinated USF-1. Huh7 cells were transfected with a plasmid that expressed the hemagglutinin (HA)-tagged ubiquitin and then infected with HCV. These HCV-infected cells were then treated with dimethyl sulfoxide (DMSO) or MG132. USF-1 was then immunoprecipitated with the anti-USF-1 antibody and analyzed by immunoblot analysis using the antibody specific to K48-linked ubiquitin. As shown in Fig. 4B, the treatment of HCV-infected cells with MG132 indeed increased the level of ubiquitinated USF-1.

We had also analyzed whether the HCV subgenomic RNA replicon cell line that expressed only the NS3-NS5B sequence could induce the degradation of USF-1 and the expression of A20 by using the HCV replicon cell line that we had previously established (12). As shown in Fig. 4C, the HCV subgenomic RNA replicon was not able to suppress the expression of USF-1 and induce the expression of A20, indicating that the induction of degradation of USF-1 via the ubiquitin-proteasome pathway by HCV likely required its complete genome.

Interestingly, as shown in Fig. 4A, the reduction of A20 by MG132 was associated with the reduction of the HCV core protein level, indicating a possible role of A20 in promoting HCV replication.

### A20 enhances HCV infection.

**FIG 5:**
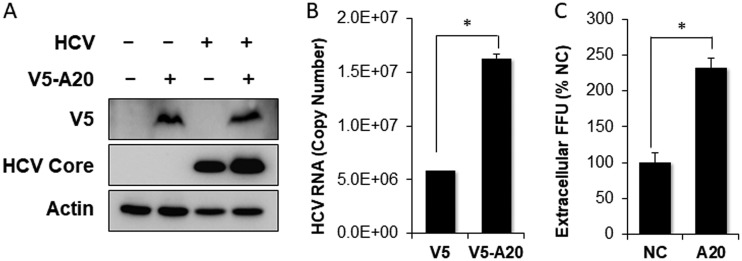
Enhancement of HCV replication by overexpression of A20. Huh7 cells were transfected with the negative-control pIRES-V5 vector or the pIRES-V5-A20-flag plasmid followed by HCV infection. Cells were lysed 48 h postinfection. (A) Immunoblot analysis of ectopically expressed A20 and the HCV core protein. (B) qRT-PCR analysis of intracellular HCV RNA. (C) Titration of released progeny HCV. The incubation media of HCV-infected cells were collected at 48 h postinfection. The viral titers, in focus-forming units (FFU), were determined by infecting naive Huh7 cells, which were then fixed and stained with the anti-HCV core antibody for immunofluorescence analysis. The results represented the average from 3 independent experiments. *, *P* < 0.05.

To examine whether A20 indeed promoted HCV replication, we transfected Huh7 cells with the A20 expression plasmid and then infected the cells with HCV. Compared with the control, the overexpression of A20 increased the HCV core protein level (Fig. 5A). The quantification of the HCV RNA in infected cells by real-time RT-PCR also revealed an increase of the HCV RNA in cells transfected by the A20-expressing plasmid (Fig. 5B). These increases in HCV core protein and RNA levels were correlated with an increase of the progeny HCV titers released from HCV-infected cells (Fig. 5C), which was determined by the focus-formation assay, indicating that the overexpression of A20 could stimulate HCV replication.

**FIG 6:**
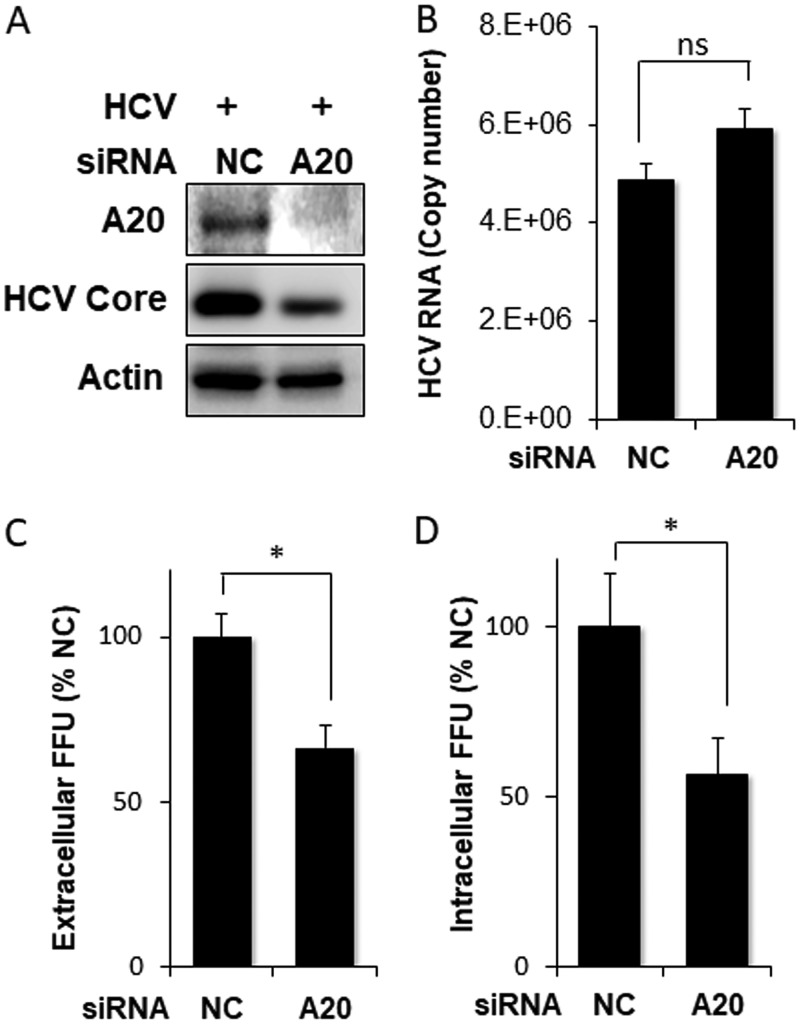
Suppression of HCV replication by A20 silencing. Huh7 cells were transfected with scrambled negative-control siRNA (NC) or the A20 siRNA and then infected with HCV for 48 h. (A) Immunoblot analysis of A20 and HCV core protein. (B) qRT-PCR analysis of intracellular HCV RNA. (C) Quantification of extracellular progeny HCV titers. The incubation media were collected at 48 h postinfection for the focus-formation assay as mentioned in the Fig. 5 legend. (D) Quantification of intracellular HCV titers as described in Materials and Methods. *, *P* < 0.05; ns, not significant.

To further determine whether A20 indeed played a positive role in HCV infection, we examined the effect of A20 knockdown on HCV replication. Huh7 cells were transfected with a control small interfering RNA (siRNA) or the A20 siRNA and then infected with HCV. As shown in Fig. 6A, the A20 siRNA efficiently reduced the A20 protein level. It also reduced the HCV core protein level. This result is in support of a positive role of A20 in HCV replication. Interestingly, the intracellular HCV RNA level was only marginally affected by A20 knockdown (Fig. 6B). As the HCV core protein is a major viral structural protein, its reduced level would be expected to negatively affect the production of progeny virus particles. That was indeed the case; as shown in Fig. 6C and D, both extracellular and intracellular progeny HCV titers, as determined by the focus-formation assay, were reduced by A20 knockdown.

### A20 promotes HCV replication by enhancing the HCV IRES activity.

**FIG 7:**
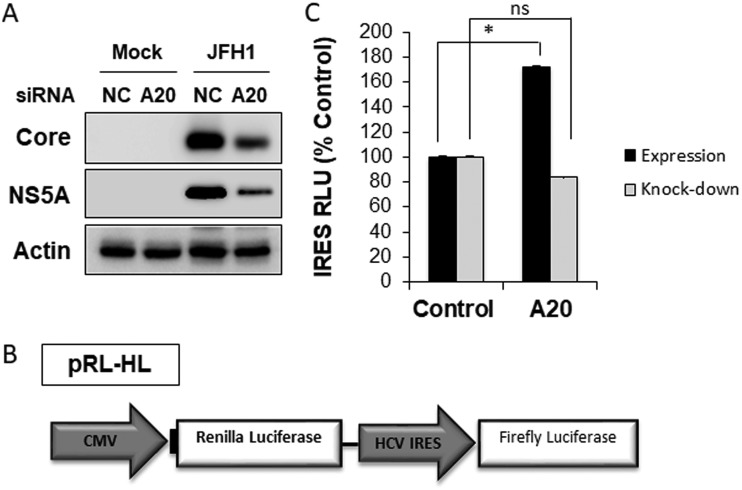
Analysis of the effect of A20 on HCV IRES. (A) Immunoblot analysis of HCV core and NS5A proteins. Huh7 cells were transfected with the control siRNA or the A20 siRNA and then infected with HCV for 48 h. Cells were then lysed for immunoblot analysis. (B) Illustration of the pRL-HL plasmid. This plasmid carries a bicistronic RNA that encodes both renilla and firefly luciferases. The translation of the renilla luciferase reporter is cap dependent, and that of the firefly luciferase is mediated by the HCV IRES. (C) Huh7 cells were transfected with pRL-HL for 48 h, after which luciferase activities were determined by the dual-luciferase assay. The firefly luciferase activity was normalized against the renilla luciferase activity, which was not significantly affected by either the overexpression or the silencing of A20. Black bars, cells were transfected with the control vector or the A20-expressing plasmid together with pRL-HL; gray bars, cells were transfected with the control siRNA or the A20 siRNA prior to the transfection with pRL-HL. *, *P* < 0.05; ns, not significant.

The A20 silencing reduced not only the HCV core protein level but also the NS5A protein level (Fig. 7A). This plus the marginal effect of A20 silencing on the intracellular HCV RNA level (Fig. 6B) prompted us to examine the possible effect of A20 on the HCV IRES, which mediates the translation of HCV proteins. We employed the plasmid pRL-HL, which expressed a bicistronic RNA encoding both the renilla luciferase and the firefly luciferase (Fig. 7B) (13). The translation of the upstream renilla luciferase is initiated by the cap-dependent mechanism and served as the internal control, whereas the translation of the downstream firefly luciferase is mediated by the HCV IRES. The cotransfection of the A20-expressing plasmid with pRL-HL into Huh7 cells increased the activity of HCV IRES, which was determined by calculating the ratio of the firefly luciferase activity to the renilla luciferase activity (Fig. 7C). In contrast, the A20 knockdown led to only a slight reduction of the HCV IRES activity. The lack of prominent effect of A20 knockdown on the HCV IRES probably was due to the low basal expression level of A20 in Huh7 cells. Nevertheless, the results shown in Fig. 7 support the argument that the upregulation of A20 by HCV could stimulate the HCV IRES activity to enhance its protein translation and hence viral replication.

## DISCUSSION

The ubiquitin-editing enzyme A20 has anti-inflammatory and immune suppressive functions (for reviews, see references 14 and 15). A20 provides negative feedback regulation of TNF-α signaling. It is induced by NF-κB after its activation by TNF-α (16), but then it mediates K63 deubiquitination of RIP1, RIP2, TRAF6, and NEMO to suppress the TNF-α signaling. We had previously demonstrated that HCV infection of hepatocytes could induce the expression of TNF-α, which then activated TNFR1 to prevent HCV from depleting IFNAR2 to sensitize HCV-infected cells to type I interferons (7). Due to the importance of A20 in the control of TNF-α signaling, we investigated the possible effect of HCV on A20. Our results indicated that HCV could transcriptionally induce the expression of A20 after infection (Fig. 1 and 2B). Interestingly, NF-κB appeared to play a minor role in the induction of A20 by HCV, as the removal of NF-κB binding sites in the A20 promoter reduced but did not abolish the effect of HCV on the A20 promoter (Fig. 2C). In contrast, mutations in the overlapping E-box and ELIE motif of the A20 promoter abolished mostly the stimulating effect of HCV on the A20 promoter (Fig. 2D). The E-box/ELIE in the A20 promoter is recognized by USF-1, a transcription factor that suppresses the A20 promoter (10, 11). Our further analysis indicated that HCV could induce the degradation of USF-1 via the ubiquitin-proteasome pathway (Fig. 3 and 4). How HCV triggered the proteasomal degradation of USF-1 is unclear. Our results indicated that the HCV subgenomic RNA replicon that did not express the structural proteins was insufficient to induce the degradation of USF-1 (Fig. 4C), suggesting the possible involvement of HCV structural proteins in this process. An earlier gene profiling report indicated that the expression of the HCV core protein by itself could induce the expression of A20 (17). However, we were not able to confirm this earlier report (data not shown). It is likely that additional HCV factors may also be required. For example, HCV has been shown to induce endoplasmic reticulum (ER) stress (12), which could trigger ER-associated proteasomal protein degradation (ERAD). Thus, it is likely that HCV-induced ER stress may also be required for the degradation of USF-1.

USF-1 is a ubiquitous transcription factor that belongs to the basic helix-loop-helix leucine-zipper (bHLH-LZ) family. Its binding site E-box, which has the sequence CACGTG, is widely distributed in the genomes of different mammalian species (18). USF-1 is involved in the transcriptional activation of PTEN (19) and Src-suppressed C kinase substrate (SSeCKS) (20), two different tumor suppressor genes. Indeed, the deletion of the E-box in the PTEN gene was found to be associated with Cowden syndrome (19), a hereditary cancer syndrome with increased risk for a variety of cancers and benign neoplasia (21). USF-1 had also been shown to stabilize the tumor suppressor p53 to promote its cell cycle arrest activity in response to DNA damage (22). The loss of USF-1 was strongly related to cell proliferation, genome instability, and tumorigenesis (22–24). Thus, it is conceivable that the depletion of USF-1 by HCV from its host cells may play an important role in HCV pathogenesis and carcinogenesis.

The induction of A20 is not unique to HCV and had also been reported before for other viruses. It had been shown that the NS1 protein of influenza A virus (IAV) could induce the expression of A20 to suppress the expression of interferon-stimulated genes (ISGs) and promote the replication of IAV (25). Similarly, it had also been reported that human cytomegalovirus (HCMV) could induce the expression of A20 by activating its promoter via the NF-κB binding sites in the early stage of infection, and the silencing of A20 suppressed the HCMV growth (26). In contrast, poliovirus had been shown to also induce the expression of A20, which suppressed the replication of the virus, indicating that the induction of A20 was an antiviral response mounted by the host cell (27). In the case of HCV, we found that A20 played a positive role in HCV replication, as overexpression of A20 enhanced HCV replication whereas its depletion had the opposite effect (Fig. 5 and 6). It will be interesting to determine whether the induction of A20 by HCV also affects interferon production or interferon signaling and thereby contributes to HCV persistence.

We had further investigated how A20 enhanced HCV replication and demonstrated that A20 could stimulate the HCV IRES activity (Fig. 7). How A20 stimulates the HCV IRES activity is yet to be elucidated. The HCV IRES is highly structured and can bind to the 40S ribosomal subunit and eIF3, which then recruit ${\text{Met-tRNA}}_{\text{1}}^{\text{Met}}$ via either the eIF2-dependent or independent mechanism to the initiation codon of the HCV RNA (5, 28). Once ${\text{Met-tRNA}}_{\text{1}}^{\text{Met}}$ is in place, the 60S ribosomal subunit is recruited to form the 80S complex for translational elongation. A20 may affect any of these assembly steps of the translation initiation complex, such as by increasing the recruitment efficiency of different factors to the HCV IRES. Further studies will be required to thoroughly understand this process. The HCV IRES belongs to the class 3 IRESs (28). It will also be interesting to determine whether this effect of A20 on HCV IRES is specific to HCV or whether it also impacts other classes of IRESs.

## MATERIALS AND METHODS

### Cell cultures and HCV preparation.

Huh7 cells were maintained in Dulbecco’s modified Eagle’s medium (DMEM) supplemented with 10% fetal bovine serum (FBS). Huh7.5 cells were maintained in DMEM supplemented with 10% FBS and 1% nonessential amino acids. The stable HCV subgenomic RNA replicon cells had been described before (12). The HCV JFH1 (HCV genotype 2a) strain variant, which produced a high level of infectious virus particles (29), was propagated in Huh7.5 cells and used in our infection studies.

### Transfection of plasmids and siRNAs.

The V5-tagged A20 expression plasmid was a gift from Pinghui Feng (University of Southern California) (11). A20-Luc-WT, A20-Luc-mNFκB, and A20-Luc-mELIE (m1) were obtained from Preet Chaudhary with the permission of Rivka Dikstein from Israel (11, 30). The pRL-HL plasmid was obtained from Stanley M. Lemon (13). The siRNAs targeting A20 were purchased from Sigma-Aldrich and transfected with Lipofectamine 2000 or Lipofectamine RNAiMax.

### Analysis of A20 promoter activity.

The analysis of the A20 promoter was conducted using the firefly luciferase reporter. The plasmid containing the renilla luciferase linked to the CMV promoter was used for the cotransfection to monitor the transfection efficiency. The luciferase activities were measured using the dual-luciferase reporter assay (Promega) according to the manufacturer’s protocol. The firefly luciferase activities were normalized against the renilla luciferase activities.

### Immunoblot analysis and antibodies.

Cells were lysed in M-PER mammalian protein extraction reagent (Thermo Scientific) with the protease inhibitor cocktail, 1 mM phenylmethylsulfonyl fluoride (PMSF), 1 mM sodium orthovanadate, and 1 mM sodium fluoride for 10 min on ice followed by a brief sonication. Cell lysates were cleared by centrifugation at 14,000 × *g* for 2 min. The supernatant was collected, boiled in Laemmli buffer for 5 min, and used for immunoblot analysis or stored at −80°C for future use. The rabbit anti-HCV core antibody was prepared in our laboratory (31). The antibody to actin was from Sigma-Aldrich, and A20 and USF-1 antibodies were from Santa Cruz. V5 antibody and horseradish peroxidase (HRP)-conjugated goat anti-rabbit and rabbit anti-mouse secondary antibodies were purchased from Abcam.

### HCV titration by focus-formation assay.

The extracellular HCV particles were harvested from the incubation medium. The intracellular HCV particles were isolated by repeated freezing and thawing of HCV-infected cells. Briefly, cells were trypsinized and then subjected to 5 cycles of freezing at −80°C followed by thawing at 37°C. Cell lysates were then centrifuged at 4,000 rpm for 5 min in a microcentrifuge to remove cell debris. The supernatant, which contained intracellular HCV particles, was collected. To perform the focus-formation assay, naive Huh7 cells on chamber slides were incubated with HCV for 20 h. The cells were then fixed with cold acetone and stained by sequential incubations with the anti-core antibody and the fluorescein-conjugated secondary antibody. The HCV core-positive cells were visualized and quantified with a Keyence Biorevo BZ9000 fluorescence microscope. The intensity of fluorescence was measured by BZII analysis software.

### Quantification of HCV RNA and analysis of gene expression.

For the quantification of HCV RNA, total cellular RNA was subjected to real-time RT-PCR using the TaqMan EZ RT-PCR kit (Applied Biosystems, Foster City, CA) according to the manufacturer’s instructions. HCV JFH1 primers 5′-TCTGCGGAACCGGTGAGTA-3′ (forward) and 5′-TCAGGCAGTACCACAAGGC-3′ (reverse) and the probe 5′-CACTCTATGCCCGGCCATTTGG-3′ were used for the qRT-PCR. The control glyceraldehyde-3-phosphate dehydrogenase (GAPDH) primer set with the probe was purchased from Applied Biosystems. For detection of other gene expressions, 100 ng total RNA was analyzed using the Power SYBR green RNA-to-CT one-step kit (Applied Biosystems, Foster City, CA). Relative RNA levels were determined after normalization against the GAPDH mRNA. The primers used are shown in Table 1.

**TABLE 1:** List of RT-qPCR primers used in TNFAIP3 studies

Gene and direction^*a*^	Primer sequence
GAPDH F	ACAACTTTGGTATCGTGGAAGG
GAPDH R	GCCATCACGCCACAGTTTC
TNFAIP1 F	ACCTCCGAGATGACACCATCA
TNFAIP1 R	GGCACTCTGGCACATATTCAC
TNFAIP2 F	GGCCAATGTGAGGGAGTTGAT
TNFAIP2 R	CCCGCTTTATCTGTGAGCCC
TNFAIP3 F	TCCTCAGGCTTTGTATTTGAGC
TNFAIP3 R	TGTGTATCGGTGCATGGTTTTA
USF-1 F	TCCCAGACTGCTCTATGGAGA
USF-1 R	CGGTGGTTACTCTGCCGAAG

a F, forward; R, reverse.

### Chromatin immunoprecipitation assay (ChIP).

Huh7 cells were collected and counted, and 4 × 10^6^ cells per sample were fixed with formaldehyde for 10 min at room temperature, followed by the addition of glycine. Cells were then lysed with the Abcam buffer with protease inhibitors and sonicated for 1 min on ice 5 times. About 15% of the cell lysates was saved to serve as the input control, and then each sample was equally separated into 4 tubes (∼1 × 10^6^ cells each). Each tube was rocked overnight after the addition of 2 μg anti-H3 histone, anti-USF-1, anti-SPT-5, or control IgG. Protein A beads were then added, and the samples were rocked for an additional 2 h. Reverse cross-linking was performed on DNA slurry (Abcam kit) by incubation with proteinase K for 30 min at 55°C and 10 min at 98°C followed by centrifugation. PCR was performed using Promega GoTaq mix for 30 cycles and analyzed by gel electrophoresis in a 2% agarose gel. The primers used were CAGCCCGACCCAGAGAGTCAC (forward) and CTCCGGGCCCCGCGATCC (reverse), which generated a DNA product approximately 300 bp in length. Quantification measurements were performed with ImageJ, and the results were normalized against the input control.

### Analysis of USF-1 ubiquitination.

Huh7 cells were transfected with the HA-tagged ubiquitin expression plasmid. Six hours later, the transfected cells were infected with HCV at an MOI of 1. At 42 h postinfection, cells were treated with MG132 for an additional 6 h, lysed with the lysis buffer (2% SDS, 150 mM NaCl, 10 mM Tris-HCl, pH 8.0, 2 mM sodium orthovanadate, 50 mM sodium fluoride, and protease inhibitors), and then boiled for 10 min. After a brief sonication, cell lysates were diluted with the dilution buffer (10 mM Tris-HCl, pH 8.0, 150 mM NaCl, 2 mM EDTA, 1% Triton) and incubated at 4°C for 30 to 60 min with rotation. After centrifugation at 20,000 × *g* for 30 min, the supernatants were collected. Equal amounts of proteins were used for immunoprecipitation using the anti-USF-1 antibody conjugated to agarose (Santa Cruz; sc-390027 AC). The antibody specific for K48-linked ubiquitin (Cell Signaling Technologies) and the USF-1 antibody (Santa Cruz) were used for immunoblotting to detect the ubiquitinated USF-1.
